# Identification of Necroptosis-Related miRNA Signature as a Potential Predictive Biomarker for Prognosis and Immune Status in Colon Adenocarcinoma

**DOI:** 10.1155/2022/9413562

**Published:** 2022-08-27

**Authors:** Xiaomei Ma, Baoshun Yang, Hexiang Dong, Hao Lin, Weigang Wang

**Affiliations:** ^1^The First Clinical Medical College, Lanzhou University, Lanzhou 730000, China; ^2^General Surgery Ward 5, First Hospital of Lanzhou University, Lanzhou 730000, China

## Abstract

**Objective:**

Increasing studies suggest that necroptosis is correlated with tumor progression. And aberrant microRNA (miRNA) expression plays a vital role in various tumors. Thus, we are committed to exploring a necroptosis-associated miRNA signature to serve as a prognostic biomarker in colon adenocarcinoma (COAD). *Data Sources and Methods.* In the current study, The Cancer Genome Atlas (TCGA) database was used to download the miRNA and mRNA expression profiles and clinical information of samples. All patients were stochastically assigned to TCGA-train and TCGA-test clusters. Subsequently, we established a prognostic signature comprised of necroptosis-related miRNAs (NR-mis) via LASSO-Cox regression and then developed a nomogram signature composed of the prognostic signature and clinical factors. Corresponding prognostic values were evaluated. Functional analysis, tumor microenvironment (TME), and chemosensitivity of risk subgroups were also identified.

**Results:**

The prognostic signature based on miR-141-3p, miR-148a-3p, miR-16-5p, and miR-200a-5p was closely associated with overall survival (OS) of samples and tumor metastasis in COAD. The Area Under Curve (AUC) was 0.605, 0.721, and 0.752 in TCGA-train cluster , 0.661, 0.613, and 0.695 in the TCGA-test cluster at 1, 3, and 5 years, respectively. The C-index for nomogram signature was 0.754. Functional analysis showed the remarkable enrichment of the signature-dependent miRNAs in tumor progression and immune response. And two risk subgroups were correlated with the distinct immune infiltration and immune checkpoints. In addition, the high-risk subgroup is more sensitive to cisplatin, doxorubicin, etoposide, and gemcitabine.

**Conclusions:**

Necroptosis-related miRNAs play a crucial role in the prognosis, metastasis, immune status, and drug sensitivity in COAD.

## 1. Introduction

As the primary cause of cancer-associated deaths globally, colon cancer ranks fourth in incidence and fifth in mortality among all tumors [1]. With an aging and growing population, economic development, and the westernization of diet and lifestyle, colon cancer has an increasing incidence [2, 3]. The majority of colon cancers are colon adenocarcinomas (COAD) originating from epithelial cells of the colon mucosa [4]. Approximately 50%–60% of patients diagnosed with colon and rectal cancer develop metastasis, and 80%–90% of these patients have unresectable metastatic liver disease, which is the main cause of most deaths [5]. Although extensive research and clinical trials have been conducted on the diagnosis and treatment of colon cancer, the therapeutic capacity of existing treatment methods is still very low [6]. The OS rate for 5 years in patients with colon cancer undergoing chemotherapy is only 10% [7]. So, it is urgent to understand the pathogenesis of colon cancer and identify predictive biomarkers to distinguish patients with high metastasis and death risk, which may contribute to future individualized treatment strategies and improve the survival outcomes of patients.

Necrosis was initially thought to be the uncontrolled random death of cells. Nowadays, it is reported that necroptosis, termed “programmed necrosis,” can be inducible and proceed regularly [8]. Necroptosis is a kind of lytic, inflammatory cell death, which is usually characterized by morphological features of necrosis, such as plasma membrane rupture, edema of cytoplasm and organelles, and overflow of cell components in the microenvironment [9, 10]. Necrosis can be caused by TNF receptor superfamily, T cell receptor, interferon receptor, Toll-like receptor, cell metabolism and genotoxic stress, or stimulation of various anticancer drugs [11, 12]. The activation of receptor-interactingserine-threonine kinase 1 (RIPK1) and receptor-interactingserine-threonine kinase 3 (RIPK3) phosphorylates the executioner molecule mixed lineage kinase domain-like (MLKL) to generate cell membrane rupture, which represents the initial stage of necroptosis [12]. Then necrotic cells release immunogenic cellular content, including damage-associated molecular patterns (DAMPs), IL-1*β*, and high mobility group box-1 (HMGB1), to trigger extreme proinflammatory processes [10, 13]. There are plenty of negative regulatory factors for necroptosis, including caspase-8 and Fas-associated protein with death domain (FADD) [12]. Inactive caspase-8 or FADD and aberrant RIPK3 activation in intestinal epithelial cells could cause intestinal inflammatory disease [14, 15].

Recent studies show that necroptosis is related to tumor progression, metastasis, and immune monitoring [16]. However, the relationships between necroptosis and cancer prognosis are not particularly clear. For that necroptosis has both antitumorigenic and protumorigenic roles [17]. Downregulation of expression of several key necroptosis mediators in cancer has been reported, such as CYLD, MLKL, and RIPK3. Low expression of MLKL protein was found to be associated with reduced overall survival in patients with colon, stomach, and pancreatic cancers [18]. Reactivation of these down-regulated necroptosis factors may be a potential anticancer therapy [19]. Necroptosis was an essential mechanism to restrict tumor metastasis [11]. For example, shikonin has been found to significantly reduce lung metastasis of osteosarcoma by inducing RIP1- and RIP3-mediated necroptosis [11]. Resibufogenin was found to inhibit the growth and metastasis of colorectal cancer by RIP3-dependent necroptosis [20]. However, studies have also shown that necroptosis can promote cancer. For example, necroptosis was found to promote pancreatic cancer cell metastasis by inducing macrophage-induced adaptive immune suppression [21]. And the necroptosis of cancer cells in the primary tumor can drive the metastasis and survival of adjacent cancer cells by inducing an inflammatory environment, attracting immune cells, or releasing cytokines [18]. For example, human and mouse melanoma cells induce endothelial cell necroptosis, promoting tumor exudation and metastasis through amyloid precursor protein (APP) and its receptor DR6.

As endogenous ∼22 nt RNA, miRNAs play important regulatory roles in gene expression by recognizing homologous sequences and interfering with transcription, translation, or epigenetic processes [22, 23]. Cellular miRNAs are involved in many important cellular activities, such as cell growth, differentiation, development, and apoptosis [23, 24]. *Cancer* especially has been the hotspot of miRNA research. More and more studies have shown that microRNAs play an important role in tumor biology, promoting tumor growth, invasion, angiogenesis, and immune escape [25], indicating their potential functions as diagnostic, prognostic, and predictive biomarkers [26]. Moreover, increasing research show that miRNAs participate in the necrotic pathway via targeting key regulators such as MLKL and RIP3 in all kinds of cancers, including colon cancer [27].

As mentioned above, we know that necroptosis is necessary in tumor development and progression. In this study, necroptosis-related miRNAs were employed to develop a prognostic signature in COAD, whose predictive ability was then verified. And the different performance of tumor microenvironment (TME) and chemosensitivity between the high-risk (HR) and low-risk (LR) subgroups were explored.

## 2. Materials and Methods

### 2.1. Data Acquisition and Preprocessing

TCGA-COAD dataset including miRNA sequencing profile of 450 COAD tissues and 8 normal colon tissues and clinical information of samples were download from The *Cancer* Genome Atlas (TCGA). Then miRNA expression data was integrated with clinical information of all samples. All the normal patients and tumor patients without overall survival time or survival status were removed, and 433 COAD patients were obtained, which all served as the TCGA-COAD cluster. Then we stochastically grouped 217 patients as a TCGA-train cluster and 216 patients as a TCGA-test cluster with the “caret” R package [28].  Table 1 presents the clinical characteristics of the samples. Moreover, the mRNA sequencing profile in the TCGA-COAD dataset was also prepared for further functional analysis.

### 2.2. Identification of Differentially Expressed Necroptosis-Related miRNAs (DE-NR-mis)

NR-mis were obtained from a prior review [29]. “Limma” package [30] was used to identify the differentially expressed NR-mis (DE-NR-mis) between tumor and normal tissue (false discovery rate (FDR) < 0.05) and “pheatmap” package was employed to visualize [31]. Then correlation network of DE-NR-mis was plotted using the R package “reshape2” [32] and “igraph” [33]. The relationship between patients' survival time and DE-NR-mis was evaluated with Univariate and Multivariate Cox regression analysis.

### 2.3. Development and Evaluation of the Prognostic Signature

LASSO Cox regression analysis was performed on candidate miRNAs to verify hub miRNAs and generate a prognostic signature using “glmnet”[34] and the “survival” package [35]. The formula is as shown as follows:

(1) $$\begin{array}{c} \text{risk score}=\Sigma Xi*Yi. \end{array}$$

*X* is the expression of miRNA and *Y* is the regression coefficient. The patients with COAD were grouped into high-risk (HR) and low-risk (LR) subgroups according to the median risk score value. The “Rms” package was utilized plot a nomogram diagram. The distribution of risk subgroups was analyzed by t-SNE and PCA approaches using the “Rtsne” [36] and “ggplot2” [37] packages. Overall survival time (OS) of samples was present on the basis of Kaplan–Meier (KM) survival analysis with the “survival” package. ROC curves, as well as their areas under the curve (AUC), were employed to assess the specificity and sensitivity of the prognostic signature using the “timeROC” package [38]. Calibration curves were applied via the “rms” package to assess the alignment between predicted survival and actual survival. The risk heatmap plotted by “limma” and the “pheatmap” packages showed the expression level of four prognostic miRNAs and the distribution of clinical characteristics between the subgroups. Finally, univariate and multivariate Cox regression analyses were performed to evaluate the correlation between patients' survival and the risk score as well as clinical characteristics.

### 2.4. Nomogram Establishment and Comparison with Other Signatures

A nomogram signature consisting of the clinical factors and the prognostic signature was developed to assess patients' outcomes. Then we randomly selected a ferroptosis-related signature [39], a pyroptosis-related signature [40] and an autophagy-related signature [41] associated with COAD on PubMed. To evaluate the prognostic value of each signature, Harrell's concordance index (C-index) and the restricted mean survival (RMS) curve analysis were performed with the “survcomp” package [42]. The C-index is the probability that the predicted outcomes are in accordance with the actual observed outcomes of samples. RMS was the expectation of life at 10 years of patients and was equivalent to the area under the survival curve. The performance of the signatures was assessed by the ratio of RMS time between the HR and LR subgroups. A greater prognostic difference can be represented by a higher RMS time ratio [43].

### 2.5. Gene Set Enrichment Analysis

To further analyze the biological functions altered by the prognostic signature in COAD, we used “clusterProfiler” and “enrichplot” [44] packages to perform GSEA enrichment analysis on the mRNA expression profiles of patients in the HR and LR subgroups, with genesets “c2.cp.kegg.v7.4.symbols.gmy” and “c5.go.v7.4.symbols.gmt” as the reference. The functions enriched by the top-ranked genes in the HR and LR subgroups were detected, respectively. The statistical threshold was FDR <0.05.

### 2.6. Target Genes Prediction and Annotation

To clarify the possible molecular functions of miRNAs that constitute the prognostic signature, we predicted target genes on the miRwalk website [45]. Only overlapped targets experimentally validated as well as predicted by TargetScan and miRDB were selected. For selected targets, Kyoto Encyclopedia of Genes and Genomes (KEGG) and Gene Ontology (GO) analysis were performed with the “clusterProfiler” and visualized with the “ggplot2” package. The GO functional analysis included molecular function (MF), cellular components (CC), and biological processes (BPs). Moreover, DO analysis was also performed using the “clusterProfiler” [46] and “DOSE” [47] packages. Then, KM survival analysis was performed on all target genes, and a gene prognostic model was established based on target genes related to OS of samples, using the same method as above.

### 2.7. Tumor Microenvironment (TME) Analysis

The “estimate” [48] package was used to calculate TME scores. Accordingly, the relationships between the risk score and TME scores were tested. Then single-sample gene set enrichment analysis (ssGSEA) was performed with the “GSVA” [49] package to compare immune cell infiltration and immune function in risk subgroups. Potential immune checkpoints retrieved from previous studies were employed to explore the connections between immune check points-related genes and risk score [50–52]. Then the relationship between the risk score and several key immune regulators was evaluated.

### 2.8. Effect of the Prognostic Signature on Sensitivity to Chemotherapy

We calculated the half maximum inhibitory concentration (IC50) of six chemotherapeutic drugs using the “pRRophetic” package [53], including cisplatin, doxorubicin, etoposide, cytarabine, mitomycin C, and gemcitabine. The IC50 of the HR and LR subgroups were compared based on the Wilcoxon signed-rank test.

### 2.9. Statistical Analysis

DEGs were identified using an empirical Bayesian approach with a threshold of FDR <0.05. LASSO COX regression analysis was used to select variables based on penalty method and construct a penalty function to obtain a more refined model. The Cox proportional-hazards model was applied to calculate the risk score [54]. The Kaplan–Meier analysis and the two-sidedlog-rank test were used to estimate the overall survival rate. Correlation analysis were analyzed using the Pearson and Spearman method. The Kruskal–Wallis test and the Wilcoxon test were used for comparative studies. Data analysis in this study was conducted with the corresponding package in *R* software. Cytoscape was used to visualize network diagrams. *P* values < 0.05 were considered significant, unless otherwise specified.

## 3. Results

### 3.1. Identification of Candidate Prognostic NR-mis

**Figure 1:**
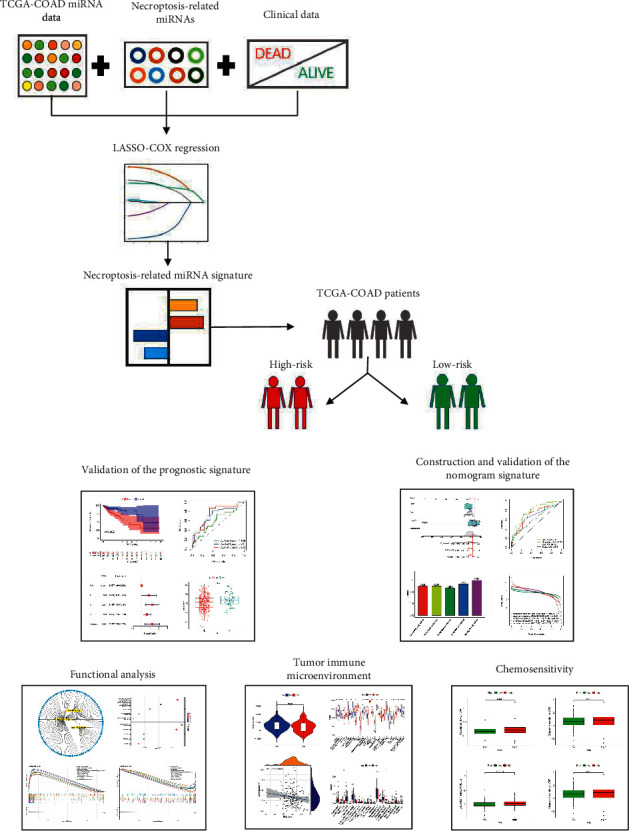
The graphical abstract of the current study.

**Figure 2:**
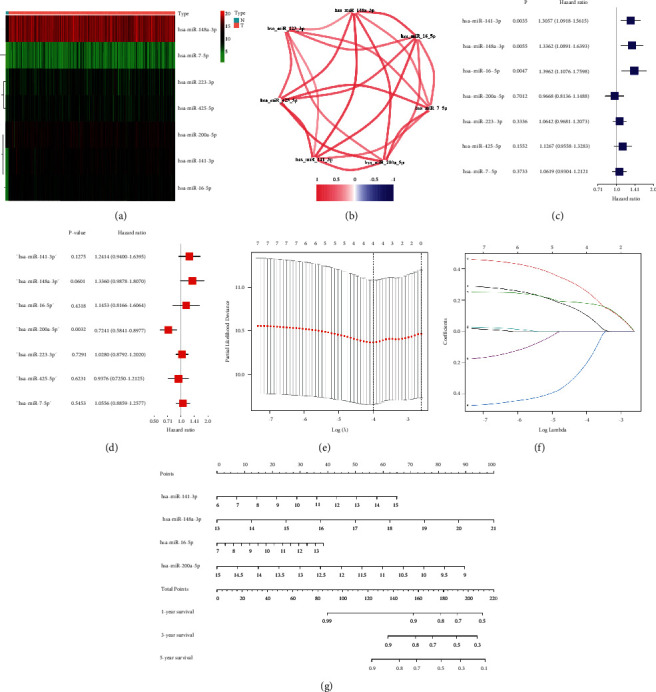
Identification of differentially expressed necroptosis-related miRNAs and construction of the prognostic signature. (a) Heatmap of the differentially expressed necroptosis-relatedx miRNAs. (b) The relational network diagram of the differentially expressed necroptosis-related miRNAs. (c, d) Significance and hazard ratio (95% CI) values of differentially expressed necroptosis-related miRNAs in univariate Cox regression (c) and multivariate COX regression (d). (e) LASSO COX regression of the 4 differentially expressed necroptosis-related miRNAs. (f) Plots of the cross-validation error rates. (g) The nomogram for prognostic signature.

A graphical abstract for this study is demonstrated in Figure 1. Totally, thirteen NR-mis were retrieved from prior review [29], nine of which exist in the TCGA-COAD miRNA expression profile (miR-331-3p, miR-148a-3p, miR-7-5p, miR-141-3p, miR-425-5p, miR-200a-5p, miR-223-3p, miR-16-5p, miR-500a-3p). The expression abundance of these miRNAs between the tumor and normal tissues was compared (Table 2), and seven DE-NR-mis were identified that were all upregulated in tumor tissues (Figure 2(a)). The correlation network showed that DE-NR-mis had a positive correlation with each other in COAD (Figure 2(b)). Then seven DE-NR-mis were analyzed by univariate and multivariate COX regression, hsa-miR-141-3, hsa-miR-148-3p and hsa-miR-16-3p had prognostic value as hazard factors in univariate COX regression analysis (Figure 2(c)), while hsa-miR-200-3p had prognostic value as a protective factor in multivariate COX regression analysis (*P* < 0.05) (Figure 2(d)). These NR-mis have been found relevant to cancer metastasis in previous studies [29]. To prevent omissions, DE-NR-mis were all considered as candidate prognostic miRNAs.

### 3.2. Development of the NR-mis Prognostic Signature in the TCGA-Train Cluster

A LASSO Cox regression analysis was utilized to construct the prognostic signature based on DE-NR-mis in the TCGA-train cluster (Figures 2(e) and 2(f)). miR-141-3p, miR-148a-3p, miR-16-5p, and miR-200a-5p were ultimately included. The final formula is shown as follows:

(2) $$\begin{array}{c} \text{risk score}=\left(Xhsa-miR-141-3p\times 0.1034\right)+\left(Xhsa-miR-148a-3p\times 0.2562\right)+\left(Xhsa-miR-16-5p\times 0.1769\right)+\left(Xhsa-miR-200a-5p\times -0.2019\right). \end{array}$$

Using the same equation, the risk scores in the TCGA-test cluster were also calculated. According to the median risk score, the samples were divided into HR and LR subgroups. The predictive process of the signature is visualized by a nomogram (Figure 2(g)).

### 3.3. Assessment of the Prognostic Signature

**Figure 3:**
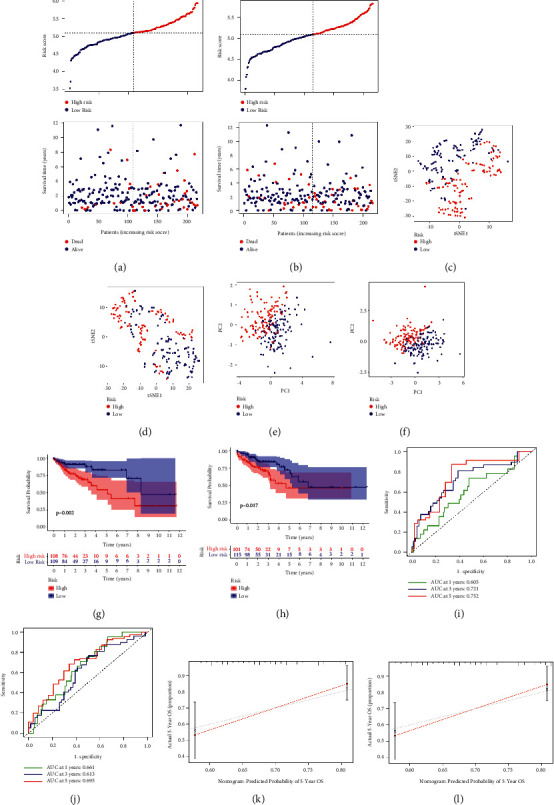
The prognostic signature assessment in TCGA-train and TCGA-test cluster. a-b Distribution diagram of risk score and survival status for samples in TCGA-train cluster (a) and TCGA-test cluster (b). PCA plot for TCGA-train cluster (c) and TCGA-test cluster (d). t-SNE analysis for TCGA-train cluster (e) and TCGA-test cluster (f). Kaplan–Meier curves of overall survival time between low-risk and high-risk subgroups in TCGA-train cluster (g) and TCGA-test cluster (h). ROC curve for measuring the predictive value at 1, 3, and 5 years in TCGA-train cluster (i) and TCGA-test cluster (j). Calibration curves of the prognostic signature in TCGA-train cluster (k) and TCGA-test cluster (l).

**Figure 4:**
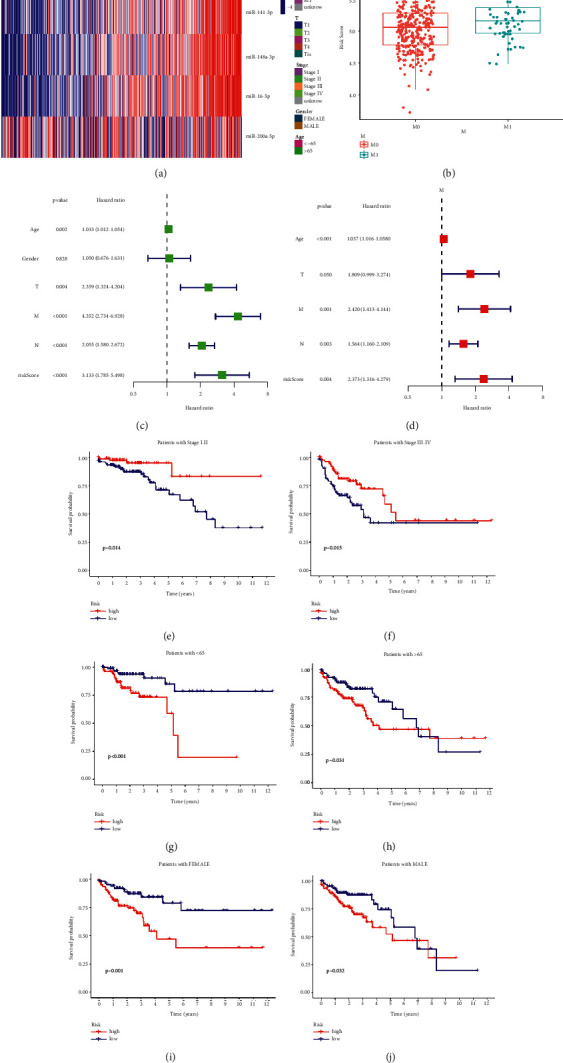
The prognostic signature assessment in TCGA-COAD cluster. (a) Heat map of miRNA expression and clinical factors between high-risk and low-risk subgroups (*p* value < 0.001, “^*∗∗∗*^”; *p* value < 0.01, “^*∗∗*^”; *p* value < 0.05,“^*∗*^”). (b) Comparison of risk scores between metastatic and nonmetastatic patients. (c) Univariate Cox regression of the prognostic signature and clinical characteristics. (d) Multivariate Cox regression of the prognostic signature and clinicopathological features. Kaplan–Meier curves for COAD patients of early stage (e), advanced stage (f), aged 65 years or younger (g), older than 65 years (h), female (i), and male (j).

We plotted the scattergram of risk score and survival status between the HR and LR groups (Figures 3(a)–3(b)). In comparison to LR group, the HR group had higher risk scores and shorter survival times. t-SNE and PCA analyses showed a clear distinction of samples in the risk subgroups (Figures 3(c)–3(f)). KM survival analysis proved the HR subgroup had a worse OS than the LR subgroup in the TCGA-train (*p*=0.002) and TCGA-test cluster (*p*=0.017) (Figures 3(g)–3(h)). The ROC-AUC at one, three, and five years was 0.605, 0.721, and 0.752 in TCGA-train cluster (Figure 3(i)), 0.661, 0.613, and 0.695 in the TCGA-test cluster (Figure 3(j)). A good consistency was observed between the predicted OS of the prognostic signature and actual OS in calibration curves (Figure 3(k)–3(l)). Subsequently, we validated the prognostic signature in the TCGA-COAD cluster. The heatmap indicated a visible difference in the metastasis rate between the HR and LR subgroups (Figure 4(a)). The risk score of metastatic samples is higher than that of without metastatic. (*p* < 0.05) (Figure 4(b)). Univariate and multivariate analyses indicated that the signature was an independent risk factor (Figure 4(c)–4(d)). Significant survival differences were also found in subgroups stratified by clinicopathological characteristics, including tumor stage, age, and gender (Figure 4(e)–4(j)).

### 3.4. Nomogram Signature Establishment and Confirmation

**Figure 5:**
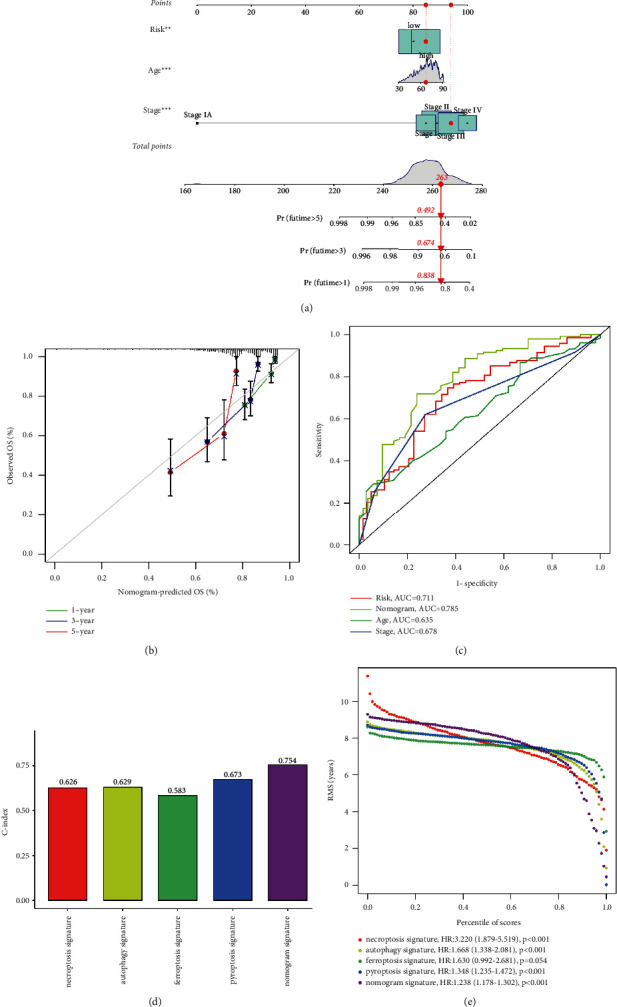
Nomogram signature construction and assessment in TCGA-COAD cluster. (a) Nomogram based on the prognostic signature and clinical characteristics to predict OS of COAD patients. (b) Calibration curves of nomogram signature. (c) ROC curve of nomogram signature, the prognostic signature and clinical characteristics for predicting OS at 5 years. (d) The C-index of necroptosis signature, autophagy signature, ferroptosis signature, pyroptosis signature and nomogram signature. (e) RMS curve of necroptosis signature, autophagy signature, ferroptosis signature, pyroptosis signature and nomogram signature. (HR = RMS time _high-risk_/RMS time _low-risk_).

Univariate and multivariate analysis showed that the age and tumor TMN stage of patients were independent risk factors in COAD (Figure 4(c)–4(d)). Hence, a prognostic nomogram signature incorporated the prognostic signature, age and tumor stage of patients was constructed (Figure 5(a)). The calibration curves show that the predicted OS of the nomogram signature has good consistency with the actual OS (Figure 5(b)). The ROC curves for 5-years showed that the nomogram signature was the most accurate for predicting COAD patients' survival rate (0.785), followed by the NR-mis signature (0.711), tumor stage (0.678) and age (0.635) (Figure 5(c)). The C-index of the ferroptosis signature, necroptosis signature, autophagy signature, pyroptosis signature and nomogram signature were 0.583, 0.626, 0.629, 0.673 and 0.754, respectively (Figure 5(d)). The nomogram signature showed satisfactory predictive capacity, suggesting that the combination of cell-death-related signature and clinicopathological characteristics can better predict patients' prognoses. RMS time ratios of five signatures between the HR and LR subgroups ranged from 1.238 to 3.220 (necroptosis signature, autophagy signature, pyroptosis signature, nomogram signature: *p* < 0.001; ferroptosis signature: *p*=0.054) (Figure 5(e)).

### 3.5. GSEA Identifies Prognostic Signature-Related Biological Functions

**Figure 6:**
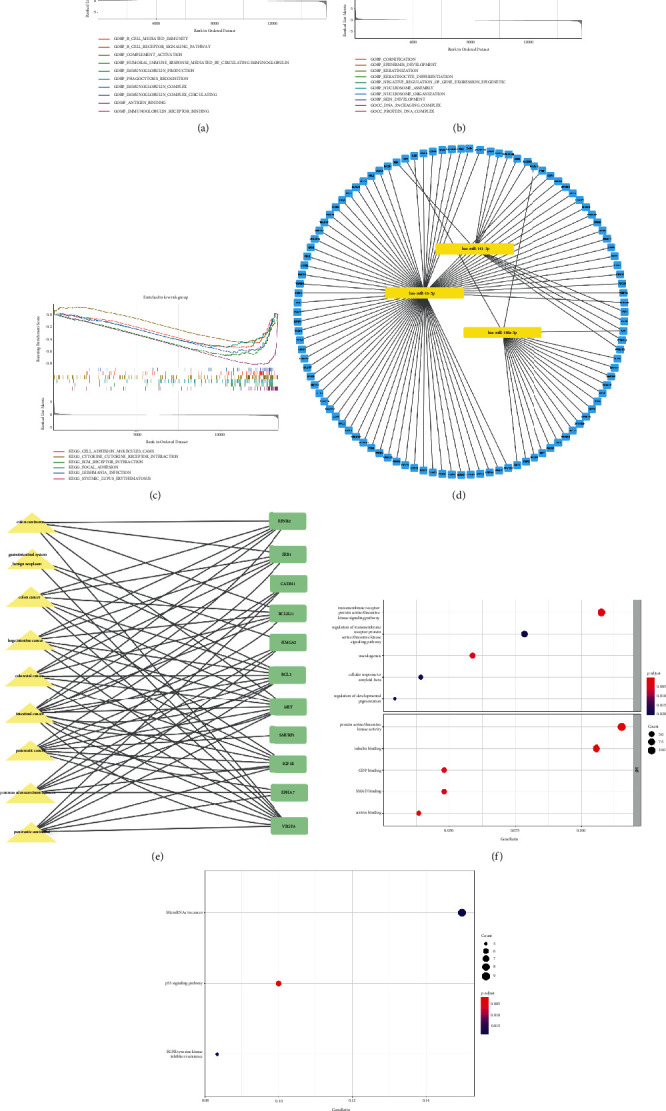
Functional analysis of the prognostic signature in TCGA-COAD cluster. (a) GSEA of top 10 enriched GO terms in the high-risk subgroup. (b) GSEA of top 10 enriched GO terms in the low-risk subgroup. (c) GSEA of 6 enriched KEGG terms in the low-risk subgroup. (d) Correlation network of prognostic miRNAs and their target genes. (e) DO analysis of gastrointestinal diseases and target genes. (f) The top 10 enriched terms in GO analysis of target genes. (g) KEGG analysis of target gene.

The purpose of GSEA analysis is to reveal the signal pathway and potential biological function of the signature in COAD. With the criterion of FDR<0.05, 15 enriched GO terms were identified in the HR subgroup, 65 enriched GO terms and 6 enriched KEGG pathways were found in the LR subgroup (Supplementary Table 1). The top enriched terms are shown in Figures 6(a)–6(c). Part of the enriched terms were closely associated with immune responses, such as humoral immune response mediated by circulating immunoglobulin, lymphocyte-mediated immunity, antigen binding, complement activation, phagocytosis recognition in the HR subgroup; and toll-like receptor signaling pathway, cytokine-cytokine receptor interaction, interferon gamma mediated signaling pathway, positive regulation of monocyte chemotaxis, positive regulation of cytokine production in LR subgroup. The results implied that the predicting effect of the prognostic signature could be related to the immune microenvironment.

### 3.6. Construction of miRNA-mRNA Network and Functional Analysis

The primary function of miRNAs is to influence gene expression mainly via recognizing the 3′untranslated region of mRNA [24]. In order to study the regulation mechanism of the NR-mis, their target genes were identified and functionally annotated. A total of 22 target genes were identified for miR-141-3p, 40 target genes for miR-148a-3p, and 156 target genes for miR-16-5p on the miRwalk website, while no overlapped target gene was found for miR-200a-5p (Supplementary Table 4). A miRNA-mRNA network was constructed by Cytoscape (Figure 6(d)). Then all target genes were annotated by GO, DO, and KEGG analysis. Do analysis indicated that 52 disease terms were related with prognostic miRNAs with a threshold of Q-value<0.05 (Supplementary Table 2), including 9 terms associated with gastrointestinal tumors (Figure 6(e)). In GO analysis, 63 enriched terms belonged to the BP category, 14 enriched terms belonged to MF category, while no significantly enriched term was found in the CC category (FDR<0.05) (Supplementary Table 3). KEGG analysis revealed enriched pathways, including microRNAs in cancer, p53 signaling, and the EGFR tyrosine kinase inhibitor resistance (FDR<0.05) (Supplementary Table 3). Top enriched terms shown in Figure 6(f) and 6(g). KM survival analysis showed that ten target genes were correlated with a patient's prognosis of COAD, including ATXN7L1, CHEK1, FKBP1A, FXR1, GALNT7, PPM1D, PRNP, SLC35D1, USP4, and VEGFA (Supplementary Figure 1). LASSO analysis confirmed that six of the target genes were hub prognostic genes. A gene prognostic model was constructed. The final formula is shown as follows:

(3) $$\begin{array}{c} \text{risk score}=\left(XATXN7L1\times 0.3959\right)+\left(XPRNP\times 0.0195\right)+\left(XGALNT7\times -0.0638\right)+\left(XFXR1\times -0.1178\right)\cdot +\left(XSLC35\;D1\times -0.2168\right). \end{array}$$

**Figure 7:**
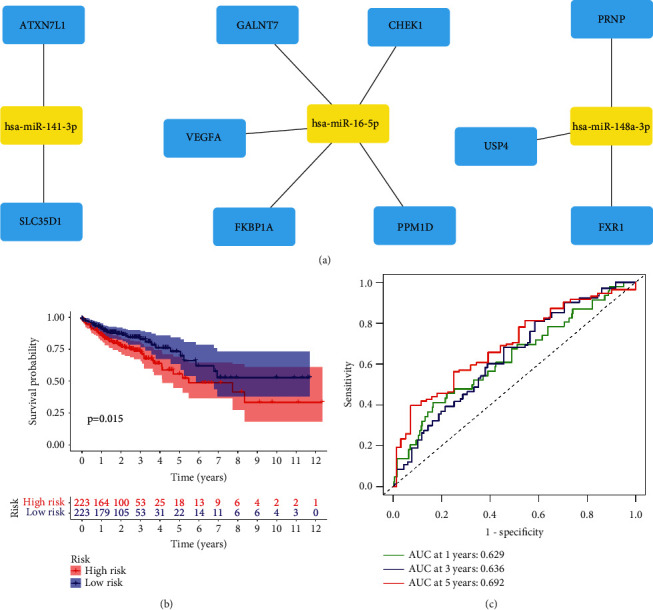
Prognostic value of target genes of necroptosis-related miRNAs. (a) Network diagram of prognostic target genes and necroptosis-related miRNAs. (b) Kaplan–Meier curves of overall survival time between low-risk and high-risk subgroups distinguished by target gene prognostic model. (c) ROC curve of target gene prognostic model for measuring the predictive value at 1, 3, and 5 years.

The corresponding relationship between prognostic model-based target genes and NR-mis was shown in Figure 7(a). The results of validation analysis showed that the prognostic model composed of the target genes of NR-mis could effectively distinguish patients in HR and LR subgroups (*p* < 0.05, Figure 7(b)). The AUC values of this model in 1, 3, and 5 years were 0.629, 0.636, and 0.692, respectively (Figure 7(c)).

### 3.7. Association between Immunity and Risk Score in COAD

**Figure 8:**
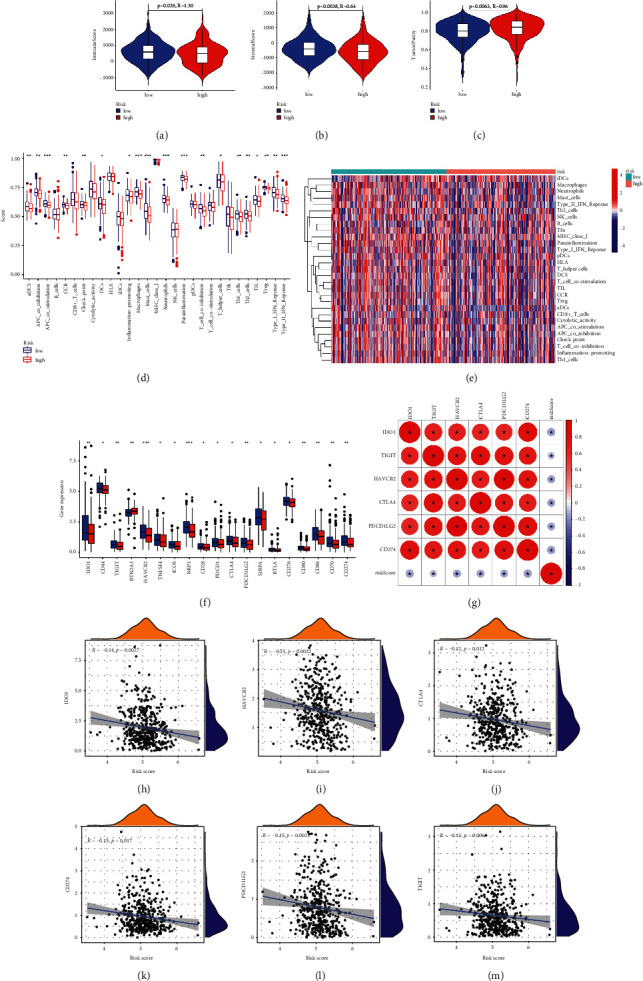
Immune-related analysis of the prognostic signature in TCGA-COAD cluster. (a–c) TME differential analysis of immune (a), stromal (b), and tumor purity (d) between high- and low-risk subgroups. (d) Comparison of the ssGSEA scores for immune cells and immune pathways between high-risk and low-risk subgroups. (e) Heatmap of immune cells and immune pathways distribution between high-risk and low-risk subgroups. (f) Expression of immune checkpoints between high-risk and low-risk subgroups. (g) Correlation analysis between risk score and six important immune checkpoint. Association between the prognostic signature and IDO1 (h), HAVCR2 (i), CTLA4 (j), CD274 (k), PDCD1LG2 (l), and TIGIT (m). (*p* value < 0.001, “^*∗∗∗*^”; *p* value < 0.01, “^*∗∗*^”; *p* value < 0.05, “^*∗*^”).

TME score showed that, taken the LR subgroup as a reference, the immune-score and stromal-score were both reduced, while tumor purity-score was increased in the HR subgroup (Figures 8(a)–8(c)). The ssGSEA analysis indicated that the HR subgroup generally had lower levels of immune cell infiltration and immune activity than the LR subgroup (Figures 8(d)–8(e)). Moreover, immune checkpoint-related genes were also lower in the HR subgroup (Figure 8(f)). The relationship between the prognostic signature and six important immune checkpoint-related genes was explored. The results showed that CD274 (PD-L1), IDO1, HAVCR2 (TIM-3), CTLA4, PDCD1LG2 (PD-L2), and TIGIT (vstm3) were all negatively related with the risk score (Figures 8(g)–8(m)), suggesting that patients in LR subgroup benefited more from immunotherapy.

### 3.8. Evaluation of Chemosensitivity in Risk Subgroups

**Figure 9:**
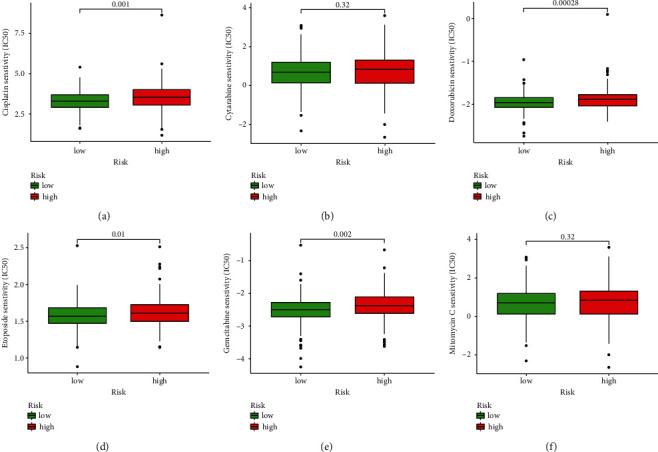
Evaluation of chemosensitivity in risk subgroups to cisplatin (a), cytarabine (b), doxorubicin (c), etoposide (d), gemcitabine (e), and mitomycin C (f).

We calculated the IC50 value to compare the chemosensitivity in two subgroups. The HR subgroup had a lower IC50 values for cisplatin, doxorubicin, etoposide, and gemcitabine, while no statistical difference was found for cytarabine and mitomycin C (Figure 9(a)–9(f)). The result indicated that samples in the HR subgroups might have higher chemosensitivity for cisplatin, doxorubicin, etoposide, and gemcitabine.

## 4. Discussion

Programmed Cell Death (PCD) has a significant impact on ontogenic development and tissue homeostasis, by means of which the organism disposes of cells that are infected, functionally dispensable, or at risk of cancerization [55]. Dysregulation of PCD is of great significance to immunological and developmental disorders, neurodegeneration, cancers and other kinds of human diseases [56]. Several types of PCD pathways, including ferroptosis, pyroptosis, and necroptosis are closely related and complementary with each other flexibly [29, 55]. Considering the important role of PCD in tumors, a growing number of studies have used PCD-related genes/noncoding RNAs to predict tumor prognosis. For example, Qi's [39] signature based on nine ferroptosis-related genes (C-index = 0.583), Wei's [40] signature based on eight pyroptosis-related genes (C-index = 0.673), and Xu's [41] signature based on eight autophagy-related genes (C-index = 0.629), all of which showed prognostic value in COAD. These and similar studies suggest that PCD-related genes/noncoding RNAs have the potential to predict tumor patients' outcomes. In this study, we extracted NR-mis miRNAs confirmed in previous research, and constructed a prognostic signature including four miRNAs and a nomogram containing the prognostic signature and clinicopathological characteristics in COAD. Our prognostic signature showed moderate predicted power. The C-index was 0.626 for the prognostic signature and 0.754 for the nomogram signature. These results confirmed the feasibility of necroptosis-related miRNAs to predict the survival outcomes of COAD patients.

The miRNAs that comprise the prognostic signature were miR-141-3p, miR-148a-3p, miR-16-5p, as well as miR-200a-5p, which have proved to regulate necroptosis in metastasis [29]. As expected, the HR and LR subgroups identified by our prognostic signature differed significantly in tumor metastasis (*P* < 0.05), and metastatic patients had a higher risk score (*P* < 0.05). Among them, miR-141-3p, miR-148a-3p and miR-16-5p were positively associated with patients' risk scores, while miR-200a-5p had a negative relationship. In other words, the superior of miR-141-3p, miR-148a-3p and miR-16-5p and the inferior of miR-200a-5p indicate a poor outcome and a high metastasis rate for COAD patients.

In order to further understand the potential function of NR-mis and their target genes in COAD, we performed functional annotations of target genes and analyzed the variable pathways between the HR and LR subgroups. Tumor metastasis involves various conditions, such as angiogenesis, the inflammatory microenvironment, the epithelial-to-mesenchymal transition (EMT), the degradation of the extracellular matrix (ECM), and programmed cell death dysfunction [11]. Interestingly, the functional annotation for the target genes of above miRNAs indicated that several pathways associated with tumor metastasis, such as TGF-*β* signaling pathway [57, 58], p53 signaling pathway [59, 60], EGFR signaling pathway [61,62], and vasculogenesis. And transmembrane receptor protein serine/threonine kinase signaling pathway and SMAD are known for involving in the activation of downstream signaling pathways of TGF-*β* [63]. Gene expression profile analysis showed that TGF-*β* signaling pathway is the most important gene pathway in liver metastasis of colorectal cancer [58]. TGF-*β* promotes cancer metastasis by stimulating EMT of tumor cells and activating angiogenesis [58]. SMAD can mediate TGF-*β*-induced EMT by inducing expression of the transcription suppressor E-cadherin [64]. The TP53 gene is mutated in 43% of colorectal cancers, and p53 function is often impaired in the remainder [65]. Mutant p53 can interact with other transcription factors, such as NF–Y, E2F1, ETS1/2, HIF-1, MED1, SMAD, and SP1, leading to COAD cell migration, metastasis, and angiogenesis [65]. Furthermore, there were also enriched pathways associated with tumor metastasis in GSEA analysis, including regulation of cell adhesion [66] and ECM receptor interaction. The results further prove that these NR-mis play a crucial part in tumor metastasis.

The effect of necroptosis on tumor progression, especially metastasis, is complex. The main regulatory factors of necroptosis have been proven to stimulate the metastasis and progression of cancer. Meanwhile, when apoptosis is damaged, necroptosis shows inhabitation of tumor development and metastasis [29]. According to a recent review, therapy-induced necroptosis is related to tumor suppression, while chronic necroptosis is relevant to metastasis [17]. Due to the insufficient blood supply, malignant tumors growing fast usually suffer from a lack of oxygen and nutrients. Necroptosis cells release cellular contents, such as HMGB1, which recruit immune inflammatory cells to liberate growth factors and prosurvival factors, prosurvival factors and proangiogenic factors to promote tumor invasion and metastasis, as well as resistance to hormonal and chemotherapy [19, 67, 68]. Chronic inflammation provoked by necroptosis can cause mutation of tumor suppressors and proto-oncogenes, thereby progressing to cancer [67]. For instance, western diet and Tsc1 ablation active aberrant necroptosis in intestinal epithelial cell, then lead to intestinal inflammation and subsequent cancer [12]. Moreover, tumor cells induce necroptosis of endothelial cells by activating death receptor 6, thus promoting extravasation and distant colonization of tumor cells [69]. On the contrary, it has been found that necroptosis has negative regulations on tumor progression. Shikonin, the first naturally occurring compound that was found to induce necroptosis, can not only highly inhibit tumor but also prevent drug resistance [70, 71]. Surviving in the circulation or a novel colonized site without interacting with the ECM, metastatic cells face numerous disadvantages, such as hypoxia, imbalance of nutrients and energy, and a lack of growth factor. In this circumstance, the activation of necroptosis triggers an ROS burst to eliminate metastatic cancer cells [11]. Overall, the effect of necroptosis on tumors is complex and varies in different circumstances. In order to better target necroptosis as tumor therapy, it is necessary to further study and clarify the different mechanisms of necroptosis on tumors.

In TME analysis, almost all immune cells, immune activities, and checkpoints were suppressed in the HR subgroup, suggesting a vital part of immunity in the anti-cancer process. In recent years, cancer immunotherapies that induce cell death instead of apoptosis have gradually emerged because quite a lot of tumors are resistant to apoptosis. Drug-induced necroptosis could inhibit tumor suppression through inducing antitumor immunogenicity [72]. In a recent study, researchers designed a nano-size “artificial necroptotic cancer cell” vaccine and vaccinated mice, causing multi-epitope-T cell responses to confront tumor cells [73]. What's more, necroptosis could cooperate with immune checkpoint blockade to suppress tumor [73]. Though relevant research is still in the initial stages, immunotherapy targeting necroptosis shows great promise.

Nevertheless, our research has some deficiencies. Firstly, miRNAs that have been identified to regulate necroptosis are relatively few so far. We are not yet able to select miRNAs of sufficient prognostic significance and in sufficient numbers to compose a more accurate prognostic signature. Hopefully, a more satisfactory prognostic signature will be established with more necroptosis-associated genes/noncoding RNAs being identified. Secondly, our conclusion was based on data mining. The function and mechanism of these miRNAs need further experimental study. And in future work, we should extend our findings to clinical cohorts.

## 5. Conclusions

Based on four necroptosis-related miRNAs, we developed a prognostic signature for COAD patients. The signature possessed a moderate predictive value of patients' outcomes. And obvious differences in metastasis rate, immune microenvironment, and chemotherapy sensitivity between the HR and LR subgroups were observed. With the further development of experimental research and the promotion of clinical trials, it is believed that necroptosis-related pathways will be of great importance in tumor diagnosis, prognosis, and treatment.

## Figures and Tables

**Table 1:** Clinical information of samples in TCGA-train and TCGA-test clusters.

Clinical characteristics	Type	Total (TCGA-COAD)	TCGA-train cluster	TCGA-test cluster	*P*.value
Age					0.302
	≤65	180 (41.57%)	96 (44.24%)	84 (38.89%)	
	>65	253 (58.43%)	121 (55.76%)	132 (61.11%)	

Gender					0.884
	Female	209 (48.27%)	106 (48.85%)	103 (47.69%)	
	Male	224 (51.73%)	111 (51.15%)	113 (52.31%)	

Stage					0.2381
	Stage I	72 (16.63%)	30 (13.82%)	42 (19.44%)	
	Stage IA	1 (0.23%)	0 (0%)	1 (0.46%)	
	Stage II	166 (38.34%)	91 (41.94%)	75 (34.72%)	
	Stage III	121 (27.94%)	57 (26.27%)	64 (29.63%)	
	Stage IV	62 (14.32%)	34 (15.67%)	28 (12.96%)	
	Unknown	11 (2.54%)	5 (2.3%)	6 (2.78%)	

T Stage					0.2744
	T1	10 (2.31%)	5 (2.3%)	5 (2.31%)	
	T2	73 (16.86%)	30 (13.82%)	43 (19.91%)	
	T3	294 (67.9%)	157 (72.35%)	137 (63.43%)	
	T4	55 (12.7%)	25 (11.52%)	30 (13.89%)	
	Tis	1 (0.23%)	0 (0%)	1 (0.46%)	

M Stage					0.5928
	M0	315 (72.75%)	158 (72.81%)	157 (72.69%)	
	M1	62 (14.32%)	34 (15.67%)	28 (12.96%)	
	Unknown	56 (12.93%)	25 (11.52%)	31 (14.35%)	

N stage					0.6187
	N0	254 (58.66%)	128 (58.99%)	126 (58.33%)	
	N1	101 (23.33%)	47 (21.66%)	54 (25%)	
	N2	78 (18.01%)	42 (19.35%)	36 (16.67%)	

**Table 2:** The expression level of necroptosis-related miRNAs between normal colon tissues and COAD tumor tissues.

Necroptosis-related miRNA	Normal tissues	Tumor tissues	logFC	FDR
miR-141-3p	4.86	11.35	1.22	3.27E-06
miR-148a-3p	12.22	17.33	0.5	3.27E-06
miR-16-5p	5.2	10.7	1.04	3.27E-06
miR-200a-5p	10.05	12.22	0.28	2.30E-05
miR-223-3p	5.67	9.53	0.75	5.07E-06
miR-331-3p	6.02	5.69	-0.08	0.269169
miR-425-5p	7.61	9.75	0.36	7.17E-05
miR-500a-3p	9.06	9.68	0.1	0.077986
miR-7-5p	1	4.95	2.31	3.27E-06

## Data Availability

The data used to support the findings of this study are included within the article.

## Supplementary Materials

B(Supplementary Materials Supplementary Table 1: enriched terms in GSEA analysis. Supplementary Table 2: enriched disease terms in DO analysis. Supplementary Table 3: enriched disease terms in KEGG and GO analysis. Supplementary Table 4: detailed information about predicted pairs of necroptosis-related miRNAs and target genes. Supplementary Figure 1: KM curves of target genes with prognostic significance. (A) ATXN7L1; (B) CHEK1; (C) FKBP1A; (D) FXR1; (E) GALNT7; (F) PPM1D; (G) PRNP; (H) SLC35D1; (I) USP4; (J) VEGFA. (K) LASSO COX regression of the target genes. (L) plots of the cross-validation error rates.
