# Supplementary material for: Identification of Necroptosis-Related miRNA Signature as a Potential Predictive Biomarker for Prognosis and Immune Status in Colon Adenocarcinoma
Source: J Oncol. 2022 Aug 27;2022:9413562. doi: 10.1155/2022/9413562 (PMC9440827; doi:10.1155/2022/9413562)
Supplement: B(Supplementary Materials — Supplementary Table 1: enriched terms in GSEA analysis. Supplementary Table 2: enriched disease terms in DO analysis. Supplementary Table 3: enriched disease terms in KEGG and GO analysis. Supplementary Table 4: detailed information about predicted pairs of necroptosis-related miRNAs and target genes. Supplementary Figure 1: KM curves of target genes with prognostic significance. (A) ATXN7L1; (B) CHEK1; (C) FKBP1A; (D) FXR1; (E) GALNT7; (F) PPM1D; (G) PRNP; (H) SLC35D1; (I) USP4; (J) VEGFA. (K) LASSO COX regression of the target genes. (L) plots of the cross-validation error rates. [file 9413562.f1.zip › Supplementary Table 1 (1).docx]

Supplementary Table 1 Enriched terms in GSEA analysis.

| Attribute | Description | FDR | rank |
| --- | --- | --- | --- |
| GOCC | DNA packaging complex | 3.03E-07 | 1031 |
| GOBP | cornification | 8.5E-06 | 503 |
| GOBP | keratinization | 8.5E-06 | 714 |
| GOBP | nucleosome assembly | 1E-06 | 1781 |
| GOCC | protein DNA complex | 1E-06 | 1330 |
| GOBP | keratinocyte differentiation | 3.7E-06 | 1344 |
| GOBP | nucleosome organization | 8.5E-06 | 2066 |
| GOBP | negative regulation of gene expression epigenetic | 1.01E-05 | 1965 |
| GOCC | cornified envelope | 0.000157 | 371 |
| GOBP | DNA heterochromatin assembly | 0.001524 | 1241 |
| GOBP | skin development | 1E-06 | 1361 |
| GOBP | DNA replication dependent nucleosome organization | 0.001109 | 571 |
| GOBP | epidermal cell differentiation | 2.23E-05 | 1344 |
| GOBP | chromatin organization involved in regulation of transcription | 0.000249 | 1965 |
| GOBP | DNA packaging | 2.16E-05 | 2066 |
| GOBP | epidermis development | 1E-06 | 1344 |
| GOBP | chromatin assembly or disassembly | 4.8E-05 | 2066 |
| GOBP | regulation of gene silencing | 0.001162 | 2078 |
| GOBP | regulation of gene expression epigenetic | 0.000311 | 673 |
| GOBP | peptide cross linking | 0.021316 | 1121 |
| GOBP | protein DNA complex subunit organization | 3.96E-05 | 2105 |
| GOBP | heterochromatin organization | 0.021547 | 1897 |
| GOBP | regulation of megakaryocyte differentiation | 0.008922 | 1759 |
| GOBP | endoderm formation | 0.017723 | 1982 |
| GOBP | megakaryocyte differentiation | 0.00382 | 1759 |
| GOBP | response to interferon gamma | 0.001698 | 2970 |
| GOBP | negative regulation of megakaryocyte differentiation | 0.036978 | 303 |
| GOMF | protein heterodimerization activity | 8.34E-05 | 1781 |
| GOBP | chromatin remodeling at centromere | 0.030125 | 1988 |
| GOBP | regulation of monocyte chemotaxis | 0.036708 | 1922 |
| GOBP | positive regulation of monocyte chemotaxis | 0.018029 | 1922 |
| GOBP | histone exchange | 0.039258 | 1988 |
| GOBP | response to zinc ion | 0.039258 | 1135 |
| GOBP | regulation of gene silencing by RNA | 0.013086 | 1759 |
| GOBP | chromatin silencing | 0.029469 | 2066 |
| GOBP | DNA replication independent nucleosome organization | 0.029469 | 3070 |
| GOBP | endoderm development | 0.047606 | 2228 |
| GOBP | regulation of leukocyte adhesion to vascular endothelial cell | 0.047622 | 1742 |
| GOBP | cellular response to zinc ion | 0.047606 | 1135 |
| GOBP | DNA conformation change | 0.000292 | 3725 |
| GOBP | interferon gamma mediated signaling pathway | 0.036978 | 3516 |
| GOBP | regulation of myeloid cell differentiation | 0.002619 | 1927 |
| GOBP | external encapsulating structure organization | 0.00069 | 2270 |
| GOBP | entry into host | 0.025265 | 2573 |
| GOBP | gene silencing | 0.001162 | 2091 |
| GOCC | external encapsulating structure | 0.000459 | 3173 |
| GOBP | negative regulation of cell activation | 0.039408 | 2587 |
| GOCC | collagen containing extracellular matrix | 0.006299 | 3200 |
| GOBP | defense response to virus | 0.021547 | 3324 |
| GOBP | leukocyte cell-cell adhesion | 0.004986 | 2309 |
| GOBP | regulation of hemopoiesis | 0.003296 | 1951 |
| GOBP | epithelial cell differentiation | 0.001162 | 2199 |
| GOBP | response to virus | 0.016497 | 3338 |
| GOMF | structural molecule activity | 0.002573 | 3009 |
| GOBP | viral life cycle | 0.029584 | 4234 |
| GOBP | myeloid cell differentiation | 0.009183 | 1937 |
| GOMF | cell adhesion molecule binding | 0.002619 | 3962 |
| GOBP | wound healing | 0.004667 | 2631 |
| GOBP | positive regulation of cell adhesion | 0.011503 | 2924 |
| GOBP | positive regulation of cytokine production | 0.023782 | 3179 |
| GOBP | positive regulation of response to external stimulus | 0.018514 | 3157 |
| GOBP | positive regulation of defense response | 0.042917 | 3782 |
| GOCC | cell substrate junction | 0.029469 | 3884 |
| GOBP | positive regulation of locomotion | 0.030125 | 3389 |
| GOBP | negative regulation of cell differentiation | 0.031547 | 2228 |
| GOBP | adaptive immune response | 0.009725 | 1172 |
| GOBP | lymphocyte mediated immunity | 0.049343 | 1379 |
| GOBP | humoral immune response | 0.029584 | 1288 |
| GOBP | regulation of B cell activation | 0.046224 | 1164 |
| GOBP | B cell mediated immunity | 0.001265 | 1491 |
| GOBP | immunoglobulin production | 0.001315 | 849 |
| GOBP | positive regulation of B cell activation | 0.005387 | 1160 |
| GOBP | complement activation | 0.00069 | 1379 |
| GOMF | antigen binding | 0.000103 | 1379 |
| GOBP | B cell receptor signaling pathway | 0.000714 | 1201 |
| GOBP | humoral immune response mediated by circulating immunoglobulin | 0.000294 | 1379 |
| GOBP | phagocytosis recognition | 0.000311 | 1160 |
| GOMF | immunoglobulin receptor binding | 8.34E-05 | 1160 |
| GOCC | immunoglobulin complex circulating | 2.82E-05 | 1160 |
| GOCC | immunoglobulin complex | 3.03E-07 | 1379 |
| KEGG | systemic lupus erythematosus | 1.83E-08 | 1253 |
| KEGG | ECM receptor interaction | 0.002339 | 3179 |
| KEGG | focal adhesion | 0.003226 | 3179 |
| KEGG | cytokine-cytokine receptor interaction | 0.033502 | 1933 |
| KEGG | cell adhesion molecules cams | 0.033502 | 2297 |
| KEGG | leishmania infection | 0.033502 | 2712 |
