# Supplementary material for: Identification of Necroptosis-Related miRNA Signature as a Potential Predictive Biomarker for Prognosis and Immune Status in Colon Adenocarcinoma
Source: J Oncol. 2022 Aug 27;2022:9413562. doi: 10.1155/2022/9413562 (PMC9440827; doi:10.1155/2022/9413562)
Supplement: B(Supplementary Materials — Supplementary Table 1: enriched terms in GSEA analysis. Supplementary Table 2: enriched disease terms in DO analysis. Supplementary Table 3: enriched disease terms in KEGG and GO analysis. Supplementary Table 4: detailed information about predicted pairs of necroptosis-related miRNAs and target genes. Supplementary Figure 1: KM curves of target genes with prognostic significance. (A) ATXN7L1; (B) CHEK1; (C) FKBP1A; (D) FXR1; (E) GALNT7; (F) PPM1D; (G) PRNP; (H) SLC35D1; (I) USP4; (J) VEGFA. (K) LASSO COX regression of the target genes. (L) plots of the cross-validation error rates. [file 9413562.f1.zip › Supplementary Table 2 (1).docx]

Supplementary Table 2 Enriched disease terms in DO analysis.

| ID | Description | Gene Ratio | qvalue |
| --- | --- | --- | --- |
| DOID:1192 | peripheral nervous system neoplasm | 12/56 | 0.005131 |
| DOID:2621 | autonomic nervous system neoplasm | 11/56 | 0.005582 |
| DOID:769 | neuroblastoma | 11/56 | 0.005582 |
| DOID:0060095 | uterine benign neoplasm | 4/56 | 0.01356 |
| DOID:13223 | uterine fibroid | 4/56 | 0.01356 |
| DOID:0060037 | developmental disorder of mental health | 10/56 | 0.01356 |
| DOID:0060086 | female reproductive organ benign neoplasm | 4/56 | 0.01356 |
| DOID:0050622 | reproductive organ benign neoplasm | 4/56 | 0.014057 |
| DOID:2316 | brain ischemia | 3/56 | 0.01609 |
| DOID:1681 | heart septal defect | 3/56 | 0.021713 |
| DOID:3908 | non-small cell lung carcinoma | 10/56 | 0.023462 |
| DOID:4074 | pancreas adenocarcinoma | 6/56 | 0.024371 |
| DOID:4045 | muscle cancer | 5/56 | 0.024371 |
| DOID:715 | T-cell leukemia | 4/56 | 0.024693 |
| DOID:0050735 | X-linked disease | 3/56 | 0.025493 |
| DOID:4905 | pancreatic carcinoma | 7/56 | 0.027548 |
| DOID:5520 | head and neck squamous cell carcinoma | 6/56 | 0.027548 |
| DOID:3910 | lung adenocarcinoma | 6/56 | 0.028555 |
| DOID:10747 | lymphoid leukemia | 4/56 | 0.028555 |
| DOID:1793 | pancreatic cancer | 8/56 | 0.028555 |
| DOID:3620 | central nervous system cancer | 5/56 | 0.029204 |
| DOID:5158 | pleural cancer | 3/56 | 0.029222 |
| DOID:7474 | malignant pleural mesothelioma | 3/56 | 0.029222 |
| DOID:2154 | nephroblastoma | 4/56 | 0.029222 |
| DOID:3565 | meningioma | 4/56 | 0.029222 |
| DOID:13025 | retinopathy of prematurity | 2/56 | 0.029469 |
| DOID:3247 | rhabdomyosarcoma | 4/56 | 0.029733 |
| DOID:633 | myositis | 4/56 | 0.031422 |
| DOID:4043 | skeletal muscle cancer | 4/56 | 0.033165 |
| DOID:5157 | benign pleural mesothelioma | 3/56 | 0.035046 |
| DOID:219 | colon cancer | 7/56 | 0.039083 |
| DOID:0050815 | eye adnexa disease | 2/56 | 0.039479 |
| DOID:9256 | colorectal cancer | 7/56 | 0.039479 |
| DOID:5672 | large intestine cancer | 7/56 | 0.039479 |
| DOID:1059 | intellectual disability | 5/56 | 0.039479 |
| DOID:201 | connective tissue cancer | 8/56 | 0.039479 |
| DOID:3602 | toxic encephalopathy | 3/56 | 0.039479 |
| DOID:0060038 | specific developmental disorder | 6/56 | 0.040093 |
| DOID:127 | leiomyoma | 4/56 | 0.040161 |
| DOID:10155 | intestinal cancer | 7/56 | 0.042585 |
| DOID:3459 | breast carcinoma | 8/56 | 0.042846 |
| DOID:50 | thyroid gland disease | 5/56 | 0.042846 |
| DOID:0050624 | gastrointestinal system benign neoplasm | 3/56 | 0.043006 |
| DOID:1542 | head and neck carcinoma | 6/56 | 0.046431 |
| DOID:423 | myopathy | 8/56 | 0.046431 |
| DOID:66 | muscle tissue disease | 8/56 | 0.046431 |
| DOID:1882 | atrial heart septal defect | 2/56 | 0.046431 |
| DOID:1520 | colon carcinoma | 5/56 | 0.046431 |
| DOID:11934 | head and neck cancer | 6/56 | 0.046431 |
| DOID:5183 | hereditary Wilms' tumor | 3/56 | 0.048303 |
| DOID:0070004 | myeloma | 7/56 | 0.049571 |
| DOID:0080000 | muscular disease | 8/56 | 0.049571 |
