# Supplementary material for: Identification of Necroptosis-Related miRNA Signature as a Potential Predictive Biomarker for Prognosis and Immune Status in Colon Adenocarcinoma
Source: J Oncol. 2022 Aug 27;2022:9413562. doi: 10.1155/2022/9413562 (PMC9440827; doi:10.1155/2022/9413562)
Supplement: B(Supplementary Materials — Supplementary Table 1: enriched terms in GSEA analysis. Supplementary Table 2: enriched disease terms in DO analysis. Supplementary Table 3: enriched disease terms in KEGG and GO analysis. Supplementary Table 4: detailed information about predicted pairs of necroptosis-related miRNAs and target genes. Supplementary Figure 1: KM curves of target genes with prognostic significance. (A) ATXN7L1; (B) CHEK1; (C) FKBP1A; (D) FXR1; (E) GALNT7; (F) PPM1D; (G) PRNP; (H) SLC35D1; (I) USP4; (J) VEGFA. (K) LASSO COX regression of the target genes. (L) plots of the cross-validation error rates. [file 9413562.f1.zip › Supplementary Table 3 (1).docx]

Supplementary Table 3 Enriched disease terms in KEGG and GO analysis.

| Attribute | Description | Gene Ratio | FDR |
| --- | --- | --- | --- |
| GO-BP | transmembrane receptor protein serine/threonine kinase signaling pathway | 11/102 | 0.004541 |
| GO-BP | vasculogenesis | 6/102 | 0.004541 |
| GO-BP | regulation of developmental pigmentation | 3/102 | 0.020234 |
| GO-BP | regulation of transmembrane receptor protein serine/threonine kinase signaling pathway | 8/102 | 0.020234 |
| GO-BP | cellular response to amyloid-beta | 4/102 | 0.020234 |
| GO-BP | regulation of microtubule polymerization or depolymerization | 5/102 | 0.020234 |
| GO-BP | coronary vasculature morphogenesis | 3/102 | 0.020234 |
| GO-BP | regulation of protein deacetylation | 4/102 | 0.020234 |
| GO-BP | regulation of microtubule cytoskeleton organization | 6/102 | 0.020234 |
| GO-BP | negative regulation of transforming growth factor beta receptor signaling pathway | 5/102 | 0.020234 |
| GO-BP | transforming growth factor beta receptor signaling pathway | 7/102 | 0.020234 |
| GO-BP | negative regulation of epithelial cell differentiation | 4/102 | 0.020234 |
| GO-BP | retinal ganglion cell axon guidance | 3/102 | 0.020234 |
| GO-BP | positive regulation of neuron death | 5/102 | 0.020234 |
| GO-BP | regulation of microtubule polymerization | 4/102 | 0.020234 |
| GO-BP | protein deacetylation | 5/102 | 0.020234 |
| GO-BP | response to amyloid-beta | 4/102 | 0.020234 |
| GO-BP | peptidyl-tyrosine phosphorylation | 9/102 | 0.020234 |
| GO-BP | peptidyl-tyrosine modification | 9/102 | 0.020234 |
| GO-BP | peptidyl-tyrosine autophosphorylation | 3/102 | 0.020234 |
| GO-BP | regulation of response to DNA damage stimulus | 7/102 | 0.020234 |
| GO-BP | transport along microtubule | 6/102 | 0.020234 |
| GO-BP | positive regulation of neuron apoptotic process | 4/102 | 0.020234 |
| GO-BP | positive regulation of protein deacetylation | 3/102 | 0.020441 |
| GO-BP | protein autophosphorylation | 7/102 | 0.020441 |
| GO-BP | protein deacylation | 5/102 | 0.022622 |
| GO-BP | positive regulation of protein serine/threonine kinase activity | 8/102 | 0.022622 |
| GO-BP | positive regulation of cell cycle | 9/102 | 0.022622 |
| GO-BP | axonal transport | 4/102 | 0.022622 |
| GO-BP | macromolecule deacylation | 5/102 | 0.023491 |
| GO-BP | regulation of protein serine/threonine kinase activity | 10/102 | 0.023806 |
| GO-BP | regulation of developmental growth | 8/102 | 0.023806 |
| GO-BP | microtubule polymerization or depolymerization | 5/102 | 0.026269 |
| GO-BP | cellular response to transforming growth factor beta stimulus | 7/102 | 0.02834 |
| GO-BP | amyloid precursor protein catabolic process | 4/102 | 0.02995 |
| GO-BP | response to transforming growth factor beta | 7/102 | 0.02995 |
| GO-BP | G2/M transition of mitotic cell cycle | 7/102 | 0.02995 |
| GO-BP | positive regulation of microtubule polymerization | 3/102 | 0.02995 |
| GO-BP | axo-dendritic transport | 4/102 | 0.03025 |
| GO-BP | stress-activated MAPK cascade | 7/102 | 0.03025 |
| GO-BP | microtubule-based transport | 6/102 | 0.03025 |
| GO-BP | regulation of cell-matrix adhesion | 5/102 | 0.031467 |
| GO-BP | regulation of mitotic cell cycle phase transition | 9/102 | 0.032284 |
| GO-BP | regulation of transforming growth factor beta receptor signaling pathway | 5/102 | 0.032284 |
| GO-BP | cytoskeleton-dependent intracellular transport | 6/102 | 0.033393 |
| GO-BP | regulation of cellular response to transforming growth factor beta stimulus | 5/102 | 0.033832 |
| GO-BP | response to radiation | 9/102 | 0.033832 |
| GO-BP | positive regulation of microtubule polymerization or depolymerization | 3/102 | 0.033832 |
| GO-BP | stress-activated protein kinase signaling cascade | 7/102 | 0.033832 |
| GO-BP | cell cycle G2/M phase transition | 7/102 | 0.033832 |
| GO-BP | negative regulation of transmembrane receptor protein serine/threonine kinase signaling pathway | 5/102 | 0.034825 |
| GO-BP | microtubule polymerization | 4/102 | 0.034825 |
| GO-BP | regulation of mRNA processing | 5/102 | 0.040068 |
| GO-BP | G2 DNA damage checkpoint | 3/102 | 0.040068 |
| GO-BP | microtubule organizing center organization | 5/102 | 0.040083 |
| GO-BP | negative regulation of microtubule polymerization or depolymerization | 3/102 | 0.041147 |
| GO-BP | mitotic G2/M transition checkpoint | 3/102 | 0.041147 |
| GO-BP | regulation of cellular response to growth factor stimulus | 7/102 | 0.043409 |
| GO-BP | regulation of cell cycle phase transition | 9/102 | 0.043409 |
| GO-BP | regulation of cell-substrate adhesion | 6/102 | 0.044631 |
| GO-BP | dephosphorylation | 9/102 | 0.049958 |
| GO-BP | regulation of MAP kinase activity | 7/102 | 0.049958 |
| GO-BP | developmental growth involved in morphogenesis | 6/102 | 0.049958 |
| GO-MF | activin binding | 4/104 | 0.00042 |
| GO-MF | protein serine/threonine kinase activity | 12/104 | 0.000851 |
| GO-MF | tubulin binding | 11/104 | 0.000851 |
| GO-MF | GDP binding | 5/104 | 0.005039 |
| GO-MF | SMAD binding | 5/104 | 0.005039 |
| GO-MF | transmembrane receptor protein kinase activity | 5/104 | 0.005041 |
| GO-MF | protein tyrosine kinase activity | 6/104 | 0.00566 |
| GO-MF | microtubule binding | 8/104 | 0.006111 |
| GO-MF | transcription corepressor activity | 6/104 | 0.021976 |
| GO-MF | phosphatase binding | 6/104 | 0.026883 |
| GO-MF | alpha-tubulin binding | 3/104 | 0.038945 |
| GO-MF | protein phosphatase binding | 5/104 | 0.041571 |
| GO-MF | transcription coregulator activity | 9/104 | 0.041571 |
| GO-MF | protein serine/threonine/tyrosine kinase activity | 3/104 | 0.046871 |
| KEGG | p53 signaling pathway | 6/60 | 0.001978 |
| KEGG | EGFR tyrosine kinase inhibitor resistance | 5/60 | 0.01842 |
| KEGG | MicroRNAs in cancer | 9/60 | 0.01842 |
