# Supplementary material for: Identification of Necroptosis-Related miRNA Signature as a Potential Predictive Biomarker for Prognosis and Immune Status in Colon Adenocarcinoma
Source: J Oncol. 2022 Aug 27;2022:9413562. doi: 10.1155/2022/9413562 (PMC9440827; doi:10.1155/2022/9413562)
Supplement: B(Supplementary Materials — Supplementary Table 1: enriched terms in GSEA analysis. Supplementary Table 2: enriched disease terms in DO analysis. Supplementary Table 3: enriched disease terms in KEGG and GO analysis. Supplementary Table 4: detailed information about predicted pairs of necroptosis-related miRNAs and target genes. Supplementary Figure 1: KM curves of target genes with prognostic significance. (A) ATXN7L1; (B) CHEK1; (C) FKBP1A; (D) FXR1; (E) GALNT7; (F) PPM1D; (G) PRNP; (H) SLC35D1; (I) USP4; (J) VEGFA. (K) LASSO COX regression of the target genes. (L) plots of the cross-validation error rates. [file 9413562.f1.zip › Supplementary Table 4 (1).docx]

Supplementary Table 4 Detailed information about predicted pairs of necroptosis-related miRNAs and target genes.

| miRNA | mRNA | Position | Score | Binding region length |
| --- | --- | --- | --- | --- |
| hsa-miR-141-3p | ZEB1 | 5UTR | 1 | 15 |
| hsa-miR-148a-3p | ZFYVE26 | 5UTR | 1 | 42 |
| hsa-miR-148a-3p | FXR1 | 5UTR | 1 | 17 |
| hsa-miR-16-5p | TLE4 | 5UTR | 1 | 26 |
| hsa-miR-16-5p | RAB30 | 5UTR | 1 | 16 |
| hsa-miR-16-5p | PPM1A | 5UTR | 1 | 16 |
| hsa-miR-16-5p | KCNN4 | 5UTR | 1 | 11 |
| hsa-miR-16-5p | RBM6 | 5UTR | 1 | 37 |
| hsa-miR-16-5p | PAFAH1B1 | 5UTR | 1 | 17 |
| hsa-miR-16-5p | TLL1 | 5UTR | 1 | 18 |
| hsa-miR-16-5p | CASK | 5UTR | 1 | 22 |
| hsa-miR-16-5p | ATXN7L3B | 5UTR | 1 | 24 |
| hsa-miR-141-3p | EPHA7 | CDS | 0.961538 | 21 |
| hsa-miR-141-3p | RAP2C | CDS | 1 | 33 |
| hsa-miR-141-3p | ZEB1 | CDS | 1 | 15 |
| hsa-miR-141-3p | PPP1R15B | CDS | 1 | 24 |
| hsa-miR-141-3p | ZEB2 | CDS | 1 | 32 |
| hsa-miR-141-3p | SLC35D1 | CDS | 1 | 17 |
| hsa-miR-148a-3p | BCL2L11 | CDS | 1 | 20 |
| hsa-miR-16-5p | PHIP | CDS | 0.961538 | 21 |
| hsa-miR-16-5p | COP1 | CDS | 0.980769 | 22 |
| hsa-miR-16-5p | CADM1 | CDS | 1 | 19 |
| hsa-miR-16-5p | SPRED1 | CDS | 1 | 13 |
| hsa-miR-16-5p | KDSR | CDS | 1 | 17 |
| hsa-miR-16-5p | CLASP1 | CDS | 1 | 10 |
| hsa-miR-16-5p | COP1 | CDS | 1 | 22 |
| hsa-miR-16-5p | COP1 | CDS | 1 | 16 |
| hsa-miR-16-5p | SLC9A6 | CDS | 1 | 13 |
| hsa-miR-16-5p | CHEK1 | CDS | 1 | 20 |
| hsa-miR-16-5p | FNDC3B | CDS | 1 | 19 |
| hsa-miR-16-5p | CPEB3 | CDS | 1 | 14 |
| hsa-miR-16-5p | SPEN | CDS | 1 | 11 |
| hsa-miR-16-5p | KIF1B | CDS | 1 | 20 |
| hsa-miR-16-5p | RAB9B | CDS | 1 | 21 |
| hsa-miR-16-5p | SMURF1 | CDS | 1 | 22 |
| hsa-miR-16-5p | ZFHX4 | CDS | 1 | 20 |
| hsa-miR-16-5p | WEE1 | CDS | 1 | 13 |
| hsa-miR-16-5p | BTAF1 | CDS | 1 | 17 |
| hsa-miR-16-5p | GTPBP1 | CDS | 1 | 16 |
| hsa-miR-16-5p | CDC23 | CDS | 1 | 10 |
| hsa-miR-16-5p | FKBP1A | CDS | 1 | 19 |
| hsa-miR-16-5p | STRADB | CDS | 1 | 21 |
| hsa-miR-141-3p | YAP1 | 3UTR | 1 | 16 |
| hsa-miR-141-3p | IGF1R | 3UTR | 1 | 15 |
| hsa-miR-141-3p | QKI | 3UTR | 1 | 16 |
| hsa-miR-141-3p | ATXN7L1 | 3UTR | 1 | 24 |
| hsa-miR-141-3p | PRELID2 | 3UTR | 1 | 23 |
| hsa-miR-148a-3p | ITGB8 | 3UTR | 0.974359 | 16 |
| hsa-miR-148a-3p | MAP3K4 | 3UTR | 1 | 19 |
| hsa-miR-148a-3p | MAP3K9 | 3UTR | 1 | 16 |
| hsa-miR-148a-3p | MAP3K9 | 3UTR | 1 | 15 |
| hsa-miR-148a-3p | DYNLL2 | 3UTR | 1 | 23 |
| hsa-miR-148a-3p | PRNP | 3UTR | 1 | 10 |
| hsa-miR-148a-3p | USP4 | 3UTR | 1 | 10 |
| hsa-miR-148a-3p | DSTYK | 3UTR | 1 | 22 |
| hsa-miR-148a-3p | DSTYK | 3UTR | 1 | 15 |
| hsa-miR-148a-3p | QKI | 3UTR | 1 | 22 |
| hsa-miR-148a-3p | DYRK1A | 3UTR | 1 | 12 |
| hsa-miR-148a-3p | ZFYVE26 | 3UTR | 1 | 20 |
| hsa-miR-148a-3p | ARL8B | 3UTR | 1 | 14 |
| hsa-miR-148a-3p | ARRDC3 | 3UTR | 1 | 15 |
| hsa-miR-148a-3p | OBI1 | 3UTR | 1 | 10 |
| hsa-miR-148a-3p | DYRK1A | 3UTR | 1 | 11 |
| hsa-miR-148a-3p | ALCAM | 3UTR | 1 | 23 |
| hsa-miR-148a-3p | MET | 3UTR | 1 | 20 |
| hsa-miR-148a-3p | RASSF8 | 3UTR | 1 | 10 |
| hsa-miR-148a-3p | TGIF2 | 3UTR | 1 | 11 |
| hsa-miR-148a-3p | BMP3 | 3UTR | 1 | 19 |
| hsa-miR-16-5p | ACVR2A | 3UTR | 0.961538 | 25 |
| hsa-miR-16-5p | PPM1D | 3UTR | 0.961538 | 23 |
| hsa-miR-16-5p | UBE2V1 | 3UTR | 1 | 23 |
| hsa-miR-16-5p | SRP72 | 3UTR | 1 | 21 |
| hsa-miR-16-5p | DICER1 | 3UTR | 1 | 17 |
| hsa-miR-16-5p | DIXDC1 | 3UTR | 1 | 37 |
| hsa-miR-16-5p | CDV3 | 3UTR | 1 | 12 |
| hsa-miR-16-5p | RAB30 | 3UTR | 1 | 12 |
| hsa-miR-16-5p | C1orf21 | 3UTR | 1 | 20 |
| hsa-miR-16-5p | PSAT1 | 3UTR | 1 | 24 |
| hsa-miR-16-5p | CCNT2 | 3UTR | 1 | 27 |
| hsa-miR-16-5p | PIP4P1 | 3UTR | 1 | 14 |
| hsa-miR-16-5p | MTMR3 | 3UTR | 1 | 22 |
| hsa-miR-16-5p | ARMCX2 | 3UTR | 1 | 10 |
| hsa-miR-16-5p | PPP6R3 | 3UTR | 1 | 16 |
| hsa-miR-16-5p | TXN2 | 3UTR | 1 | 10 |
| hsa-miR-16-5p | RPS6KA3 | 3UTR | 1 | 18 |
| hsa-miR-16-5p | TMEM154 | 3UTR | 1 | 39 |
| hsa-miR-16-5p | LUZP1 | 3UTR | 1 | 12 |
| hsa-miR-16-5p | TMCC1 | 3UTR | 1 | 24 |
| hsa-miR-16-5p | EZH1 | 3UTR | 1 | 13 |
| hsa-miR-16-5p | TSC22D2 | 3UTR | 1 | 14 |
| hsa-miR-16-5p | ZC3H11A | 3UTR | 1 | 21 |
| hsa-miR-16-5p | KIF1B | 3UTR | 1 | 19 |
| hsa-miR-16-5p | AHCYL2 | 3UTR | 1 | 16 |
| hsa-miR-16-5p | RAB9B | 3UTR | 1 | 30 |
| hsa-miR-16-5p | GALNT7 | 3UTR | 1 | 15 |
| hsa-miR-16-5p | CDC37L1 | 3UTR | 1 | 13 |
| hsa-miR-16-5p | UBFD1 | 3UTR | 1 | 11 |
| hsa-miR-16-5p | CLDN2 | 3UTR | 1 | 12 |
| hsa-miR-16-5p | TAOK1 | 3UTR | 1 | 24 |
| hsa-miR-16-5p | TAOK1 | 3UTR | 1 | 21 |
| hsa-miR-16-5p | DMTF1 | 3UTR | 1 | 23 |
| hsa-miR-16-5p | ZMAT3 | 3UTR | 1 | 28 |
| hsa-miR-16-5p | IPPK | 3UTR | 1 | 19 |
| hsa-miR-16-5p | SRPK1 | 3UTR | 1 | 18 |
| hsa-miR-16-5p | TGFBR3 | 3UTR | 1 | 40 |
| hsa-miR-16-5p | HMGA2 | 3UTR | 1 | 14 |
| hsa-miR-16-5p | EFNB2 | 3UTR | 1 | 13 |
| hsa-miR-16-5p | ARL3 | 3UTR | 1 | 21 |
| hsa-miR-16-5p | KIF3B | 3UTR | 1 | 15 |
| hsa-miR-16-5p | ONECUT2 | 3UTR | 1 | 17 |
| hsa-miR-16-5p | CAPZA2 | 3UTR | 1 | 18 |
| hsa-miR-16-5p | TBL1XR1 | 3UTR | 1 | 24 |
| hsa-miR-16-5p | FBXL20 | 3UTR | 1 | 24 |
| hsa-miR-16-5p | CCND2 | 3UTR | 1 | 30 |
| hsa-miR-16-5p | FNTA | 3UTR | 1 | 14 |
| hsa-miR-16-5p | BCL2 | 3UTR | 1 | 18 |
| hsa-miR-16-5p | SLC6A4 | 3UTR | 1 | 12 |
| hsa-miR-16-5p | CHEK1 | 3UTR | 1 | 19 |
| hsa-miR-16-5p | ARL2 | 3UTR | 1 | 15 |
| hsa-miR-16-5p | VEGFA | 3UTR | 1 | 26 |
| hsa-miR-16-5p | BACE1 | 3UTR | 1 | 20 |

“Score” refers to the ability to combine, with a maximum score of 1. The higher the score, the more possibility of the combination of the miRNA and target gene.
