# Supplementary figures and images for: Identification of Necroptosis-Related miRNA Signature as a Potential Predictive Biomarker for Prognosis and Immune Status in Colon Adenocarcinoma
Source: J Oncol. 2022 Aug 27;2022:9413562. doi: 10.1155/2022/9413562 (PMC9440827; doi:10.1155/2022/9413562)

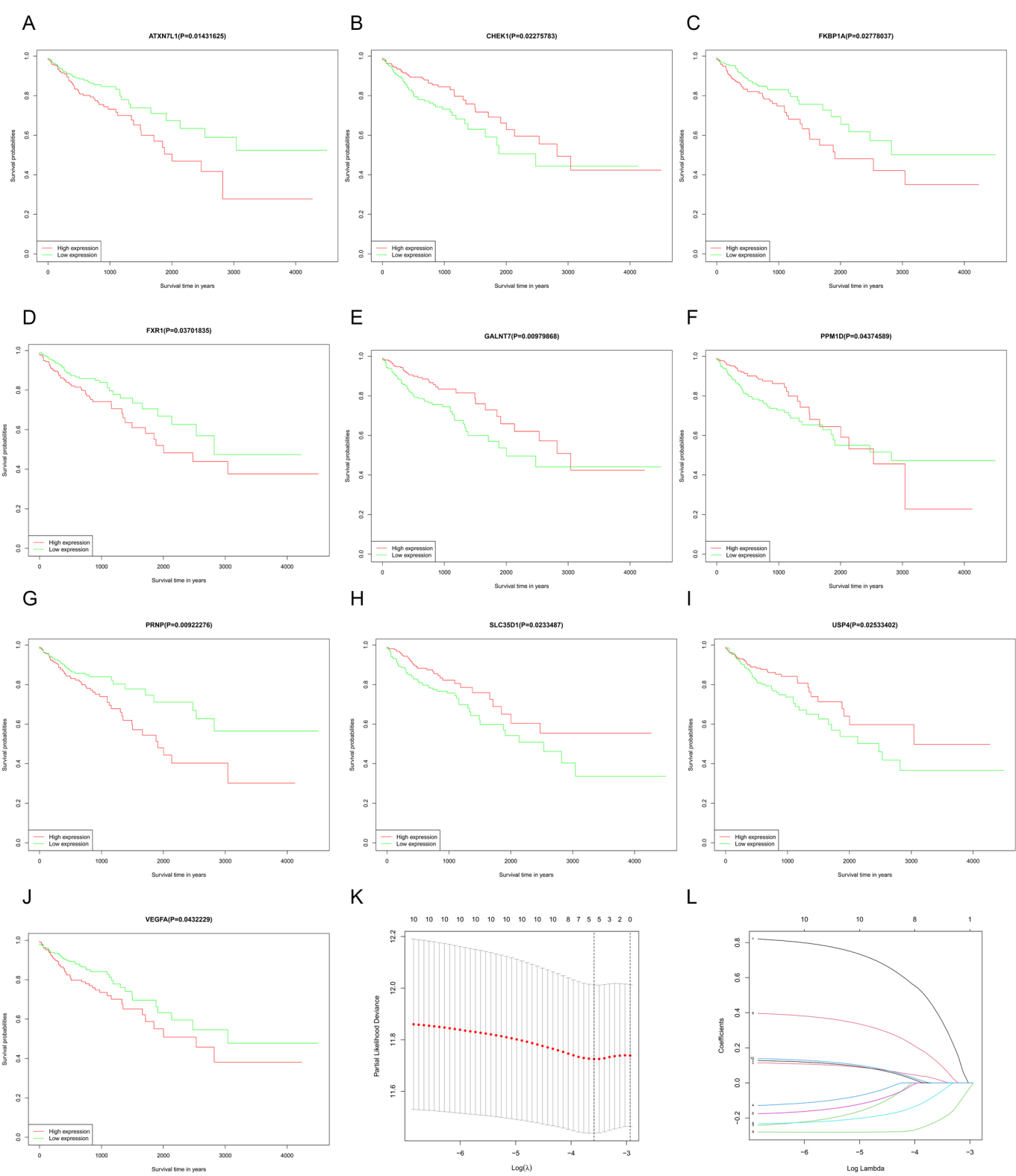

Supplement: B(Supplementary Materials — Supplementary Table 1: enriched terms in GSEA analysis. Supplementary Table 2: enriched disease terms in DO analysis. Supplementary Table 3: enriched disease terms in KEGG and GO analysis. Supplementary Table 4: detailed information about predicted pairs of necroptosis-related miRNAs and target genes. Supplementary Figure 1: KM curves of target genes with prognostic significance. (A) ATXN7L1; (B) CHEK1; (C) FKBP1A; (D) FXR1; (E) GALNT7; (F) PPM1D; (G) PRNP; (H) SLC35D1; (I) USP4; (J) VEGFA. (K) LASSO COX regression of the target genes. (L) plots of the cross-validation error rates. [file 9413562.f1.zip › Supplementary Figure 1 (1).pdf]
